# Effect of Sample Collection (Manual Expression vs. Pumping) and Skimming on the Microbial Profile of Human Milk Using Culture Techniques and Metataxonomic Analysis

**DOI:** 10.3390/microorganisms8091278

**Published:** 2020-08-21

**Authors:** Maricela Rodríguez-Cruz, Claudio Alba, Marina Aparicio, María Ángeles Checa, Leonides Fernández, Juan Miguel Rodríguez

**Affiliations:** 1 Laboratorio de Nutrición Molecular, Unidad de Investigación Médica en Nutrición, Hospital de Pediatría, Centro Médico Nacional Siglo XXI. Instituto Mexicano del Seguro Social. Av. Cuauhtémoc No. 330, Col Doctores, Delegación Cuauhtémoc, Mexico City 06725, Mexico; 2 Department of Nutrition and Food Science, Faculty of Veterinary Sciences, Complutense University of Madrid, 28040 Madrid, Spain; marinaap@ucm.es; 3 Department of Galenic Pharmacy and Food Technology, Faculty of Veterinary Sciences, Complutense University of Madrid, 28040 Madrid, Spain; claudio.alba@ucm.es (C.A.); leonides@ucm.es (L.F.); 4 Centro de Salud Arrabal, 50015 Zaragoza, Spain; manchecadiez@gmail.com

**Keywords:** skimming, human milk, culture techniques, metataxonomic analysis, microbiota, manual collection, pumps

## Abstract

Human milk microbiota is a unique bacterial community playing a relevant role in infant health, but its composition depends on different factors (woman health, lactation stage, and geographical lactation). However, information is lacking regarding some other factors that may affect the bacterial community of human milk. In this study we aimed to study the impact of the sample collection method and the skimming procedure using culture-dependent and culture-independent techniques to study the human milk microbial profile. One set of milk samples was provided by women (*n* = 10) in two consecutive days; half of the samples were collected the first day by manual expression and the other half on the second day by pumping. The rest of the participants (*n* = 17) provided milk samples that were fractionated by centrifugation; the bacterial profiles of whole milk and skimmed milk were compared by culture techniques in 10 milk samples, while those of whole milk, fat and skimmed milk were subjected to metataxonomic analysis in seven samples. Globally, the results obtained revealed high interindividual variability but that neither the use of single-use sterile devices to collect the sample nor the skimming procedure have a significant impact of the microbial profile of human samples.

## 1. Introduction

The role of human milk as a complex ecological niche and as a relevant source of bacteria to the infant gut has become evident in the last years [1]. It is known that the quantitative and/or qualitative composition of many components of human milk (peptides, proteins, lipids, immunological compounds, oligosaccharides, etc.) may be influenced by several factors, including genetic background, geographical location, maternal nutrition, part of the feeding (foremilk, hindmilk), gestational age, circadian rhythm and lactation stage. However, little is known on the interaction and impact of these and other factors on human milk microbial communities [2,3]. The modification of the milk microbiota composition may influence infant colonization resulting in a different infant gut microbiota profile. This modification could have an impact on the metabolism and, also, the development and maturation of the immune and neuroendocrine systems [4]. Due to this, it is necessary to know if these modifications are due to external factors such as the sample collection and processing of the milk for the analysis of microbiota composition.

The application of both culture-dependent and culture-independent techniques to human milk may seem a simple and straightforward approach but, in fact, the collection of a representative milk sample for the microbial analysis is not an easy issue, due to the absence of standard protocols for the collection, storage and analysis of this biological fluid. There are several sampling-related factors that may affect the result of microbiome analysis of human milk [5,6]. They include the method of expression (manual collection and pump extraction), type of pump (single use and repeated use), breast and/or pump cleaning procedures before milk expression and the milk phase (fat layer, aqueous phase or whole milk) used for the analysis. More specifically, the use of milk pumps (and other devices) to collect the samples is associated to a high concentration of contaminant bacteria (particularly enterobacteria, *Pseudomonas* spp., *Stenotrophomonas* spp. and related Gram-negative bacteria), and yeasts, that arise from the rinsing water, poor hygienic manipulations and other sources, but are not related to the milk-specific microbiota [7,8,9]. In a previous study, 60 women were recruited in order to elucidate the influence of manual milk pumps on the milk microbial load and profile. The frequency of detection and the concentration values for genera and species, which belongs to the natural microbiota of human milk (*Staphylococcus epidermidis*, *Streptococcus mitis/oralis*, *Streptococcus salivarius*, *Rothia* spp. and *Corynebacterium* spp.) were similar independently of the milk collection method [10]. In contrast, both the frequency and the mean concentration values for members of the Family Enterobacteriaceae, other Gram-negative bacteria and yeasts were significantly higher among samples obtained using the mothers’ own milk pumps than among those obtained by manual expression. Such contaminating microbial groups were also detected on the surfaces of the pump devices [10].

On the other hand, the fat fraction is typically eliminated during the first steps of the DNA extraction when human milk is submitted to microbiome analysis. Since some of the members of the human milk microbiome (including different species belonging to *Corynebacterium* and *Cutibacterium* (previously known as *Propionibacterium*)) seem to be highly lipophilic [11], the type of milk (whole milk or skimmed milk) used in microbiome studies may also affect the results of the analysis.

In this context, the aim of this study was to assess the impact of the sample collection method and the skimming procedure on the results obtained when submitting human milk samples to culture-techniques and metataxonomic analysis.

## 2. Materials and Methods

### 2.1. Participating Women and Collection of the Samples

In this study, 27 women were recruited in order to elucidate the contribution of milk extraction method and milk skimming on the microbial profile of human milk. Women with symptoms of mastitis, Raynaud’s disease or breast abscess were excluded from this study. All mothers participating in the study gave written informed consent to the protocol (reference 10/017E), which had been previously approved by the Ethical Committee of Clinical Research of Hospital Clínico San Carlos, (Madrid, Spain). The study was carried out in accordance with the Declaration of Helsinki.

**Figure 1:**
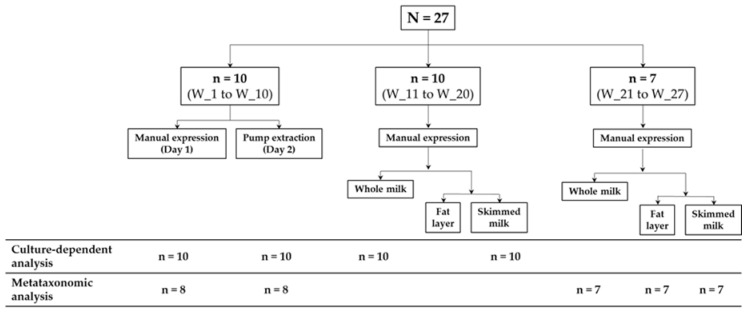
Scheme of the sampling and analysis performed in this study.

A first group of 10 women (women 1 to 10) provided two sequential milk samples; the first one was collected by manual expression (using a gloved hand) following the protocol described by Arroyo et al. [5] into single-use, sterile polypropylene milk collection containers with polybutylene terephthalate caps (Medela, Inc.; McHenry, IL, USA) (Figure 1). The following day, a second sample was collected from the same breast by using a single-use, sterile polypropylene milk collection container with a polybutylene terephthalate cap (Medela, Inc.) using an electric breast pump. There were only two exceptions (women 8 and 10), which collected their second samples with their own pumps and devices, which were routinely cleaned and rinsed with tap water at their homes.

Another group of 10 women (women 11–20) provided a complete sample (latch) of milk (breast emptying) from one of their breasts via manual expression as indicated above. For each woman, one aliquot (1 mL) of whole milk was kept for further analysis and, subsequently, and another 1-mL aliquot/the rest of the sample was centrifuged at 800× *g* for 15 min at 4 °C to separate the fat layer from the aqueous phase (Figure 1). Aliquots of whole milk and skimmed milk were used for culture-based analysis.

The last group of 7 women (women 21–27) provided the same milk sample of women 11–20 but, in this case, the sample was divided in three fractions (whole milk, fat layer and skimmed milk) and aliquots of the three fractions were used for DNA extraction and metataxonomic analysis.

All the aliquots were immediately frozen (−20 °C). In order to eliminate or minimize potential laboratorial biases, all the samples were submitted to a single freeze–thaw cycle and were analyzed by the same researchers using the same reagents’ batches and equipment.

### 2.2. Cultures and Identification of Isolates

Decimal dilutions of the milk samples (or their fractions) in sterile peptone water were prepared and plated onto Columbia Nadilixic Acid (CNA, BioMérieux Marcy l’Etoile, France; medium for the isolation of staphylococci, streptococci, enterococci, corynebacteria and related Gram-positive bacteria), MacConkey (MCK, BioMérieux; medium for the isolation of enterobacteria), Sabouraud Dextrose Chloramphenicol (SDC, BioMérieux; medium for the isolation of yeasts) and De Man, Rogosa and Sharpe (MRS, Oxoid; Basingstoke, UK) supplied with 0.05% (*w*/*v*) l-cysteine agar plates (MRS-Cys; medium for the isolation of lactic acid bacteria). CNA, MCK and SDC plates were incubated in aerobiosis at 37 °C for 48 h while MRS-Cys plates were incubated anaerobically (85% nitrogen, 10% hydrogen and 5% carbon dioxide) in an anaerobic workstation (MINI-MACS, DW Scientific, Shipley, UK) at 37 °C for 48 h. After incubation, counts in each growth medium were recorded and, subsequently, at least one representative of each colony morphology was selected from the agar plates. DNA was purified from isolates and they were identified by either 16S rDNA sequencing or matrix assisted laser desorption ionization-time of flight (MALDI-TOF) mass spectrometry using a Vitek-MS™ instrument (BioMérieux, Marcy l′Etoile, France) as described previously [12].

### 2.3. DNA Extraction from the Milk Samples

All the samples (1 mL each) were centrifuged for 15 min at 11,000× *g* at 4 °C. Extraction of DNA from the pellets was performed following the protocol of QIAamp DNA Stool Mini Kit (Qiagen, Hilden, Germany) as described by the manufacturer, with the following modifications: (a) the samples were mechanically lysed using the FastPrep-FP120 (Thermo Scientific, Waltham, MA, USA) and glass beads matrix tubes (3 cycles × 60 s, speed 6); (b) after centrifugation, the RNA from the supernatant was removed using ribonuclease A (10 mg/mL) and incubated 15 min at 37 °C and (c) the protein fraction was eliminated with a proteinase K (15 µL of a 20 mg/mL stock solution) treatment at 70 °C for 10 min. Extracted DNA was eluted in 20 µL of nuclease-free water and stored at −20 °C until further analysis. The DNA concentration was estimated using a Nanodrop ND-1000 UV Spectrophotometer (Nano-Drop Technologies, Wilmington, DE, USA). Two negative control blanks, which included no sample, were subject to all steps of the DNA extraction and purification procedure described above.

### 2.4. PCR Amplification and Sequencing

A dual-barcoded 2-step PCR reaction was conducted to amplify a fragment of the V3–V4 hypervariable region of the bacterial 16S ribosomal RNA (rRNA) gene. Equimolar concentrations of the universal primers S-D-Bact-0341-b-S-17 (ACACTGACGACATGGTTCTACACCTACGGGNGGCWGCAG) and S-D-Bact-0785-a-A-21 (TACGGTAGCAGAGACTTGGTCTGACTACHVGGGTATCTAATCC) were used as previously described [13], generating amplicons of approximately 464 bp from the V3–V4 hypervariable region. The primers were synthesized by Isogen Life Sciences (Castelldefels, Spain). Barcodes used for Illumina sequencing were appended to 3′ and 5′ terminal ends of the PCR amplicons to allow for the separation of forward and reverse sequences. A bioanalyzer (2100 Bioanalyzer, Agilent, Santa Clara, CA, USA) was used to determine the concentration of each sample. Barcoded PCR products from all samples were pooled at approximately equimolar DNA concentrations and run on a preparative agarose gel. The correct sized band was excised and purified using a QIAEX II Gel Extraction Kit (Qiagen, Madrid, Spain) and then quantified with PicoGreen (BMG Labtech, Jena, Germany). Finally, one aliquot of pooled, purified, barcoded DNA amplicons was sequenced using the Illumina MiSeq pair-end protocol (Illumina Inc., San Diego, CA, USA) at the facilities of the Scientific Park of Madrid (Spain). The sequences analyzed for this study are available in the BioSample database of the National Center for Biotechnology Information (BioProject ID PRJNA545235).

The amplified fragments and results were taxonomically analyzed using the Illumina™ software according to the manufacturer’s guidelines and pipelines (v. 2.6.2.3 San Diego, CA, USA). The resulting high-quality reads were assembled and classified taxonomically into operational taxonomic units (OTUs) by comparison with the Illumina™ software according to the manufacturer’s guidelines and pipelines (v. 2.6.2.3) using a Bayesian classification method and a level of similarity of at least 97%.

The concentration of DNA in the two blank preparations was approximately 0.01 ng/µL while that obtained from the biological samples that were included in the 16S rRNA sequencing analysis was, at least, 570 ng/µL. In addition, no amplification was detected from the blank samples after the first PCR and, as a consequence, the two blank controls were not submitted to sequencing. The decontam R package [14] was used in order to identify, visualize and remove contaminating DNA based on DNA concentration in each sample.

### 2.5. Bioinformatic and Statistical Analysis

A bioinformatic analysis was also conducted combining R (v. 3.2.3), QIIME pipelines (v. 1.8.0) [15]. Alpha diversity was assessed with the Shannon diversity index, which considers the number and evenness of microbial species. Differences in Shannon diversity indices between groups were assessed using Kruskal–Wallis tests (variables with more than 2 groups) or Wilcoxon rank tests (variables with 2 groups). Beta diversity was studied using principal coordinates analysis (PCoA) to visually display patterns of beta diversity through a distance matrix containing a dissimilarity value for each pairwise sample comparison. For the quantitative and qualitative analyses, the Bray–Curtis and binary Jaccard indices were used, respectively, followed by PERMANOVA analysis with 999 permutations to reveal statistically significant differences (*p* < 0.05). Differences in the median values of the relative abundance of bacterial phyla and genera between samples obtained using different collection methods or between different milk fractions were compared by Kruskal–Wallis tests or Wilcoxon rank tests. To correct for multiple comparisons, Bonferroni-adjusted significance levels were performed. Comparison of the frequencies of detection was performed using Fisher exact tests.

## 3. Results

### 3.1. Culture-Dependent Analysis of the Samples: Effect of the Collection Method

In order to test the effect of the collection method on the milk microbial profile, a total of 10 women provided two samples from the same breast, one collected by manual expression on the first day and by pumping on the second one, in two consecutive days. The 20 samples were plated on CNA, MRS-Cys and MCK agar plates, and bacterial growth was observed in 5 (25%), 15 (75%) and 15 (75%) samples, respectively (Table 1). No growth from any sample was observed on SDC plates, indicating the absence of yeast and molds in all the samples. The dominant bacterial species in these samples was *Staphylococcus epidermidis*. Members of *Streptococcus mitis* and *Streptococcus salivarius* groups (*Streptococcus parasanguinis/lactarius* and *Streptococcus salivarius*), and other Gram-positive bacteria (*Kocuria* spp. and *Corynebacterium* spp.), including a few lactic acid bacteria (*Lactococcus lactis*, *Ligilactobacillus salivarius* (previously known as *Lactobacillus salivarius)* and *Limosilactobacillus reuteri* (previously known as *Lactobacillus reuteri*) were also isolated.

The microbial profile found in each woman was similar, both at the concentration (ranging from 2.30 to 3.86 log_10_ cfu/mL) and at the species levels, independently of the collection method as far as the “pump” samples were collected using single-use devices (women 1–7 and 9; Table 1). In contrast, differences were found between both collection procedures when women used their own pumps (women 8 and 10; Table 1). More specifically, the bacterial counts in CNA and MRS-Cys plates (corresponding mainly to the species *S. epidermidis* and *Str. salivarius*) in the sample collected with a pump of woman 8 were at least 10 times higher in the sample obtained by manual expression (Table 1). In the case of woman 10, there was no detectable growth in any growth media when the sample was collected manually but, in contrast, a high concentration (>4 log_10_ cfu/mL) of enterobacteria (*Klebsiella oxytoca*) and water- and soil-related Gram-negative bacteria (*Stenotrophomonas maltophilia*) was observed when the sample was obtained by pumping (Table 1).

### 3.2. Culture-Dependent Analysis of the Samples: Effect of Skimming

A total of 10 different women provided one emptying sample (a complete latch) from one breast to compare the microbial profile of whole milk and skimmed milk within each woman. Among the 20 samples, growth was observed from 16 (80%) and 14 (70%) samples when they were plated on CNA and MRS-Cys agar plates, respectively. No growth from any sample was observed on MCK or SDC plates (Table 2), indicating the absence of enterobacteria and yeasts in the milk samples, since these culture media are selective for these specific groups of microorganisms, respectively. Again, *S. epidermidis* was the dominant bacterial species, while *Str. salivarius* and other Gram-positive bacteria (*Kocuria kristinae*, *Rothia mucilaginosa* and *Corynebacterium* spp.) as well as some lactic acid bacteria (*Lc. lactis*, *L. salivarius* and *Enterococcus faecium*) were only detected in some samples. The microbial load found in the whole milk samples ranged from 2.15 to 2.72 log_10_ cfu/mL and did not differ from that found in the skimmed milk samples, which ranged from 2.04 to 2.73 log_10_ cfu/mL. Only one or two bacterial species were identified from each sample, and the results identical for both whole and skimmed milk samples obtained from the same women (Table 2).

### 3.3. Microbiome Analysis of the Samples: Effect of the Collection Method

The comparison of the metataxonomic microbial profile of the samples depending on the collection method (manual expression vs. pumping with a single-use device) was performed using 8 of the 10 pairs of samples described in Section 3.1. (Figure 1). A total of 1,320,599 high quality-filtered sequences were obtained from the 16 samples analyzed in this part of the study, and the number of sequences ranged from 40,401 to 123,262 per sample (mean ± SD: 103,230 ± 12,280), and corresponded to 605 different OTUs. Samples collected either by manual expression or by pumping with a single-use sterile device were characterized by similar Shannon and Simpson diversity indices reflecting similar alpha diversity of the microbiome in the milk samples (Figure S1A).

**Figure 2:**
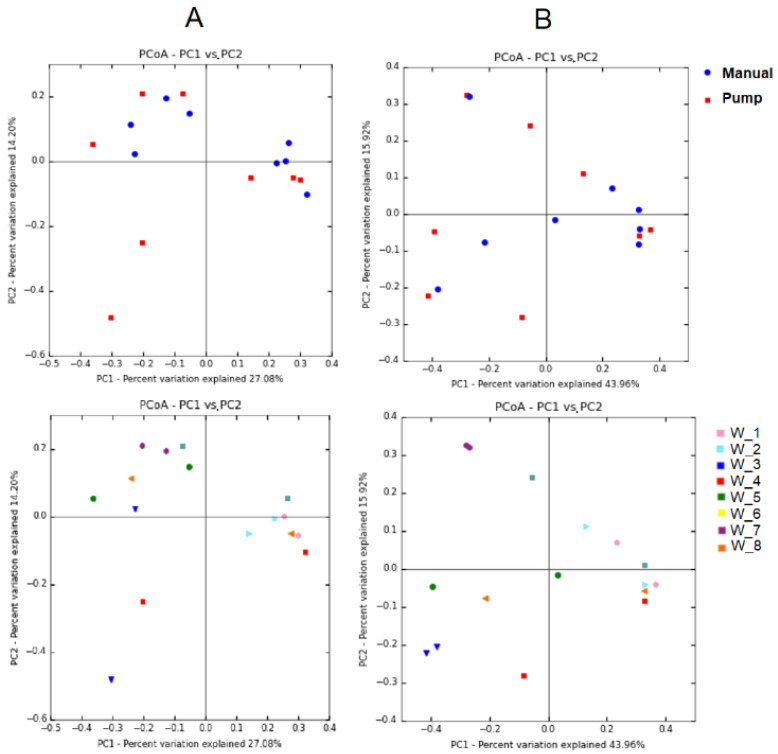
PCoA plots of bacterial profiles (at operational taxonomic unit (OTU) level) based on the Jaccard’s coefficient for binary data (presence/absence) (**A**) and on the Bray–Curtis similarity analysis (relative abundance) (**B**) of the milk samples collected from women 1 to 8. The value given on each axis label represents the percentage of the total variance explained by that axis. In the upper figures, color and shape of the symbol indicate the collection method (red squares, pump using a single-use device; blue circles, manual expression). In the bottom figures, different colors of the symbols represent the samples provided by each woman, independently of the collection method.

At the OTU level, the two-dimensional principal coordinate analysis (2D-PCoA) of the Jaccard distances enabled the visual assessment of the lack of clustering of samples according to the collection method, indicating the lack of relationship among the microbial profiles of samples collected manually and those collected using single-use pumps (Figure 2A, upper panel). The subsequent PERMANOVA analysis to test for differences in bacterial composition according to the Jaccard similarity (presence/absence of OTUs) revealed that there was no difference between the two groups of samples collected by different methods (*p* = 0.671). The PCoA plot based on the Bray–Curtis similarity, which was calculated in accordance to the relative abundances of different OTUs, also showed that the samples did not cluster according to the collection method (Figure 2B, upper panel). The PERMANOVA analysis of similarity confirmed that there was no difference between both groups of samples with regard to the bacterial community structure (*p* = 0.322). In contrast, most of the pairs of samples provided by each woman were in close proximity, indicating that the subject had a more intense influence on the milk bacterial diversity than the method used to collect the milk sample (Figure 2A and Figure 1B, lower panel). Individual differences in the microbiome composition (at the OTU level) of milk samples were statistically significant among women according to both Jaccard and Bray–Curtis similarity indices (*p* = 0.013 and *p* = 0.030, respectively; PERMANOVA).

**Figure 3:**
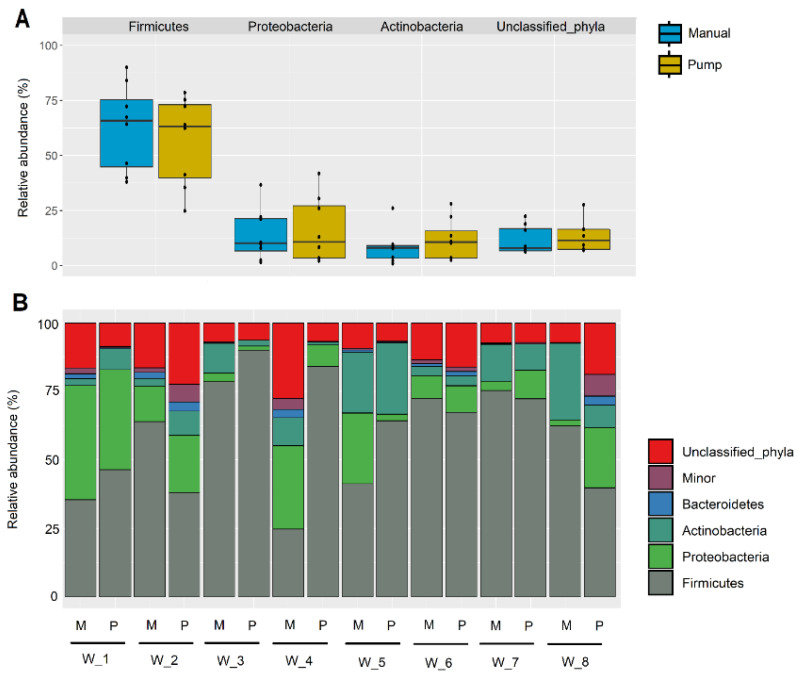
(**A**) Comparison of the relative abundance of sequences (%) belonging to main bacterial phyla obtained by metataxonomic analysis from milk samples obtained using a pumping device (blue) or by manual expression (yellow). (**B**) Individual profile of the relative abundance of the most abundant phyla in the milk samples provided by each woman (*n* = 8; W_1 to W_8) using two collection methods: manual expression (M) or using a pumping device (P).

The relative abundance of the most common phyla found in the two types of samples is presented in Figure 3. The following phyla were detected in all the samples: Firmicutes, Actinobacteria, Proteobacteria and Bacteroidetes. Firmicutes was the most abundant phyla, having median (Interquartile range, IQR) relative abundances of 63.22% (39.75–73.12%) in manually collected milk samples and 65.83% (44.70–75.26%) in samples obtained by pumping. Two other phyla, i.e., Proteobacteria and Actinobacteria, were found at similar abundance but in lower proportion than Firmicutes. The median (IQR) relative abundance of Actinobacteria was 10.58% (3.31–15.65%) and 7.96% (3.26–9.03%) in samples manually collected and obtained by pumping, respectively. Sequences belonging to unclassified phyla were also observed (Figure 3A). About 11.37% (7.31–16.39%) of the OTUs could not be assigned to any known phylum in samples manually collected and 7.86% (6.66–16.79%) in samples collected by pumping. No significant differences were found between both groups of samples at the phylum level (*p* > 0.05; Wilcoxon tests). The individual profiles of the relative abundance of OTUs at the phylum level showed that the women effect was stronger than the collection method (Figure 3B).

**Figure 4:**
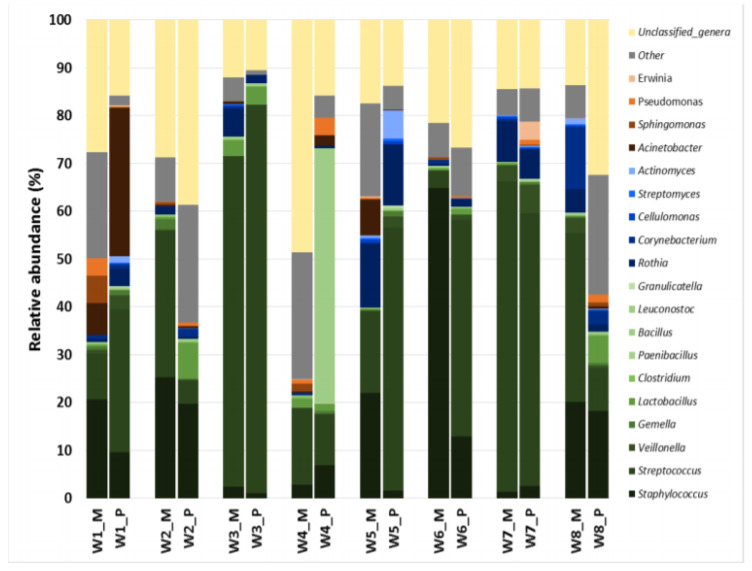
Comparison of individual profiles of the relative abundance of the most abundant bacterial genera in the milk samples provided by each woman (*n* = 8; W_1 to W_8) using two collection methods: manual expression (M) or using a pumping device (P).

In both sets of milk samples (manually or pump obtained), *Streptococcus* and *Staphylococcus* were detected in all samples and at the highest relative abundance (Table 3). The median (IQR) values of OTUs assigned to *Streptococcus* were 23.94% (14.22–42.83%) and 20.36% (2.74–22.82%), while those identified as *Staphylococcus* were 20.36% (2.74–22.82%) and 8.26% (2.37–14.22%), in samples obtained by manual expression or by pumping, respectively. *Rothia* was the third most abundant genus after *Streptococcus* and *Staphylococcus*, but its relative abundance was markedly lower (median (IQR) value of 3.27% (0.72–6.51%) in manually obtained milk samples and 1.6% (1.26–4.3%). In the rest of the genera the median values of relative abundance were lower than 0.5%. There were no differences either in the abundances of bacterial genera between samples collected by manual expression or by pumping. Additionally, the frequency of detection of the most common genera did not vary according to the collection method (Fisher exact probability tests, data not shown) The individual profiles of the relative content of the most abundant bacterial genera in the milk samples provided by each women using both collection methods is shown in Figure 4. The comparison of these profiles revealed a relatively stable microbiota structure in samples provided by the same woman that was not influenced by the extraction method.

### 3.4. Microbiome Analysis of the Samples: Effect of Skimming

In order to determine if the skimming step during sample preparation would modify the milk metataxonomic profile, samples provided by seven women (W_21 to W_27) as indicated in Section 3.1. (Figure 1) were used to prepare three fractions: whole milk, fat layer and skimmed milk. The metataxonomic analysis generated a total of 763,228 high quality-filtered sequences for the 21 samples. The number of sequences ranged from 15,540 to 121,818 per sample, which were assigned into 376 different OTUs. Overall, no significant differences were found in terms of alpha diversity (Shannon and Simpson indices) between the sample sets of whole milk, fat layer and skimmed milk samples (*p* > 0.05; Kruskal–Wallis tests; Figure S2).

**Figure 5:**
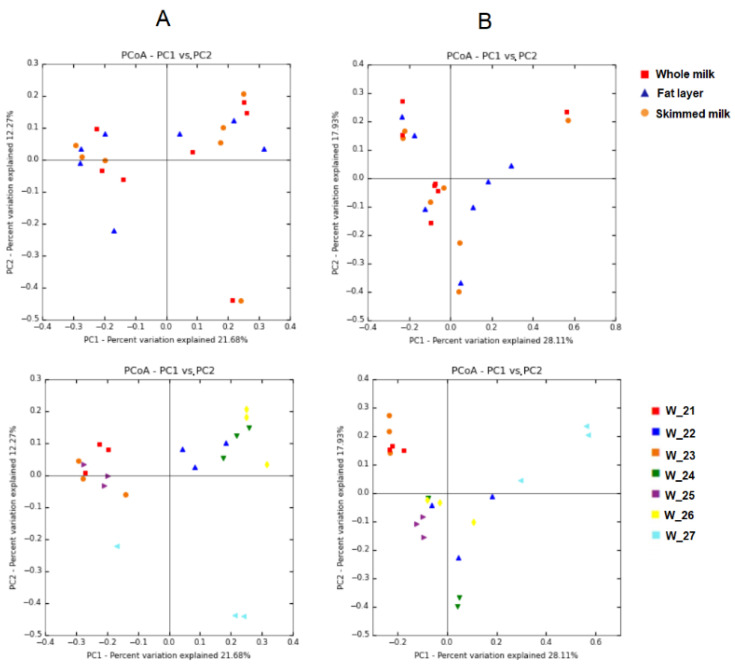
PCoA plots of bacterial profiles (at the OTU level) based on the Jaccard’s coefficient for binary data (presence/absence) (**A**) and on Bray–Curtis similarity analysis (relative abundance) (**B**) from the whole milk samples (*n* = 7) collected from women 21 to 27 and their fractions: the fat layer and the skimmed milk. The value given on each axis label represents the percentage of the total variance explained by that axis. In the upper figures, color and shape of the symbol indicate the milk fraction (red squares, whole milk; blue triangles, fat layer; orange circles, skimmed milk). In the bottom figures, different colors of the symbols represent the samples provided by each woman, independently of the milk fraction.

Potential differences in the microbiome profile of samples depending on the milk fraction (whole milk, fat layer and skimmed milk) were searched using PCoA plots of the bacterial profiles based on Jaccard’s coefficient and on Bray–Curtis index (Figure 5). Bacterial profiles of the milk samples did not cluster according to the milk fraction in the PCoA plots (Figure 5A,B upper panels). The subsequent analyses of similarity revealed that there were no differences between the three groups of fractions in relation to the presence/absence of OTUs or the relative abundance of OTUs (*p* = 0.950 and *p* = 0.840, respectively; PERMANOVA). As shown above (Section 3.3.), milk samples clustered depending on the women according to the Jaccard and Bray–Curtis indices of similarity (*p* = < 0.001 and *p* = < 0.001, respectively; PERMANOVA; Figure 5A,B, bottom panels).

**Figure 6:**
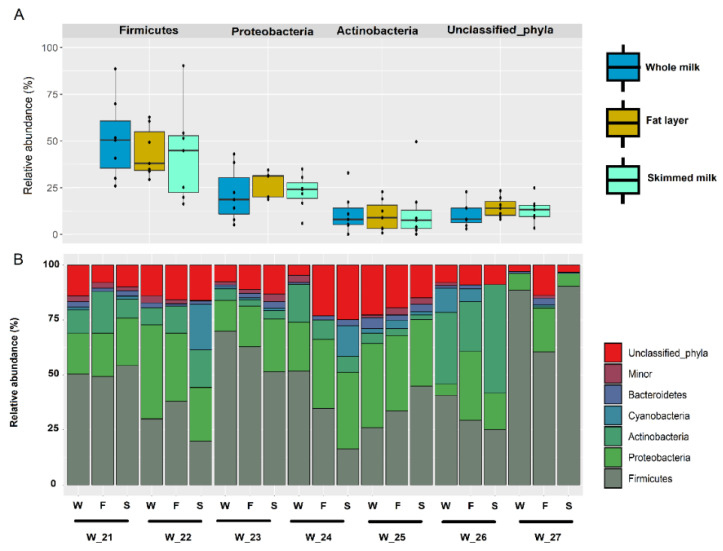
(**A**) Comparison of the relative abundance of sequences (%) belonging to main bacterial phyla obtained by metataxonomic analysis from the whole milk (dark blue) samples (*n* = 7) collected from women 21 to 27 and their fractions: the fat layer (yellow) and the skimmed milk (light blue). (**B**) Individual profile of the relative abundance of the most abundant phyla in the whole milk (W) samples provided by each woman (*n* = 7; W_21 to W_27) and their fractions: the fat layer (yellow) and the skimmed milk (light blue).

**Figure 7:**
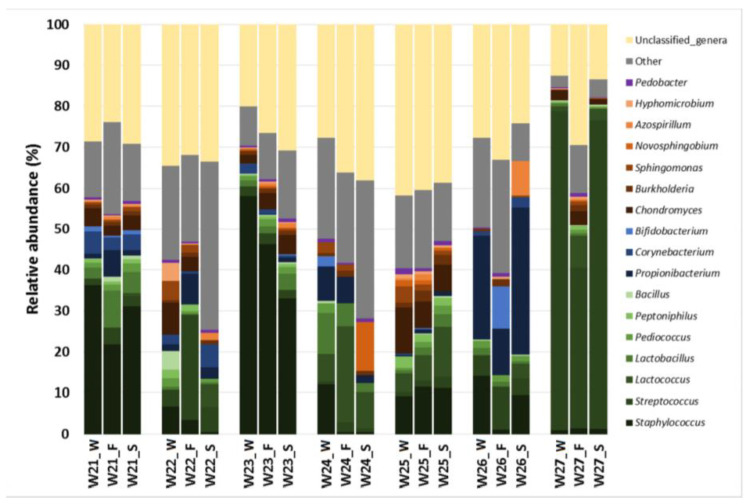
Comparison of individual profiles of the relative abundance of the most abundant bacterial genera in the whole milk (W) samples provided by each woman (*n* = 7; W_21 to W_27) and their fractions: the fat layer (F) and the skimmed milk (S).

In this set of 21 samples, the milk microbiome was also dominated by Firmicutes, which had a median (IQR) relative abundance in the three fractions similar to the values reported above, and, specifically, 50.35% (35.28–60.77%), 37.85% (34.17–54.88%) and 44.76% (22.4–52.83%) in whole milk, in the fat layer and in skimmed milk samples, respectively (Figure 6A). There were some differences regarding Proteobacteria and Actinobacteria, when these samples were compared with the previous (Section 3.3.) While the relative abundance of Proteobacteria was slightly higher (median (IQR) values of 18.53% (10.85–30.32%), 31.06% (19.86–31.41%) and 23.92% (19.15–27.45%), respectively for whole milk, fat layer and skimmed milk samples), that of Actinobacteria was lower (median (IQR) values of 7.84% (5.10–14.00%), 8.79% (3.05–15.59%) and 7.45% (3.05–12.83%) for the same set of samples; Figure 6a). The proportion of OTUs that could not be assigned to a specific phylum was also higher (Figure 6A). The comparison of the individual profiles of the most abundant phyla indicated, as above, wide interindividual differences, in contrast to the high similarity between the three fractions obtained from the sample provided by each woman (Figure 6B). Similar observations could be made regarding the comparison of the relative abundance of the most abundant genera in the set of whole milk, the fat layer and the skimmed milk fractions (Table 4) or in the individual profiles of the three fractions for each individual milk sample (Figure 7).

## 4. Discussion

In this study, two factors that may exert an influence or introduce a bias when studying the microbiota and microbiome of human milk were investigated. More specifically, the studied factors were the impact of the milk collection method (manual expression vs. pump expression) and milk skimming. Globally, our results showed differences in the individual milk microbial composition depending on each woman, due to a high interindividual variability, but not differences were noted depending on the milk collection method or the milk skimming. The results of a previous study aimed to evaluate the stability of the milk bacterial communities within women indicated that milk bacterial communities were generally complex and that the community was often stable over time within an individual and different to those present in other women [16].

For each individual woman, no differences were observed between the microbiota and microbiome of manually expressed milk samples and those of milk samples obtained by pumping using single-use devices. Such devices have been used recently to compare the milk microbiome of women living in different geographical and socioeconomical settings [17]. In contrast, there were relevant differences between the two types of collection methods in the case of the two women who were asked to use their own pumps (which were, therefore, submitted to a repeated use). In addition, no statistically significant differences were observed among the milk microbiota and microbiome of each woman depending on the milk fraction (whole milk, cream and skimmed milk) studied.

In the last 15 years, several studies have described the normal presence of viable commensal, mutualistic or potentially probiotic bacteria in human milk [1], leading to an increasing interest in the assessment of the human milk microbiota and microbiome and their functions for the maternal and/or infant health. Cultivable bacteria found in human milk are usually dominated by bacteria belonging to the genera *Staphylococcus*, *Streptococcus*, *Corynebacterium*, *Cutibacterium* and related Gram-positive bacteria [18,19,20]. At a lower extend, lactic acid bacteria (*Lactobacillus*, *Lactococcus*, *Leuconostoc*, *Ligilactobacillus*, *Limosilactobacillus*, *Weissella* and *Enterococcus*) and bifidobacteria can be also isolated from human milk [21,22,23,24,25]. Most of the bacterial isolates cultured in this study belonged to any of the genera cited above. However, the microbial pattern was quantitatively (bacterial concentration) or qualitatively (bacterial species) different for the two women who were asked to use their own pumps. Previous studies have revealed that the use of milk pumps may result in a high concentration of contaminating Gram-negative bacteria (in particular, those belonging to the Enterobacteriaceae family and to the genera *Pseudomonas* and *Stenotrophomonas*) and yeasts arising from rinsing water and/or poor hygienic manipulation practices [7,8,10]. Nevertheless, we did not observe an impact of use of milk pumps, because no differences in the microbiome milk were observed between manually expressed milk and those of milk obtained by pumping using single-use devices.

On the other hand, DNA extraction procedures for microbiome analysis of human milk involve a defatting step. Since some of the bacterial species commonly isolated from milk (such as *Corynebacterium kroppenstedtii*, *Corynebacterium tuberculostearicum*, *Corynebacterium amycolatum* or *Propionibacterium acnes*, which has been recently reclassified as *Cutibacterium acnes*) are lipophilic and may be located within lipid vacuoles [1], skimmed milk could have a different microbiome composition than whole milk. However, no differences in the microbiome of whole milk, cream or skimmed milk obtained from the same woman were observed in this study. This observation may be related to the fact that the main fat-related genera (Corynebacterium and Propionibacterium) were minority in this study and, also, to the wide intersample variability in milk bacterial composition, which could mask changes in the relative abundance of these genera in the different fractions. In addition, the fat fraction might contain residual amounts of skim milk due to the fractioning procedure used in this work. On the other hand, the membrane surrounding the milk fat globules contains membrane-specific glycosylated proteins, such as mucins, which may interact with many bacteria, including lactic acid and Gram-negative bacteria [26,27]. Therefore, the lack of differences in the microbial profile of the three milk fractions may be related to the fact that bacteria-fat interactions are a broad feature not associated with specific taxonomical units.

While it is becoming evident that human milk microbiome may be influenced by several factors and, also, that human milk microbiota may exert a strong influence on maternal/infant health, the exact triggers or drivers of differences in the composition of the human milk microbiota/microbiome need to be elucidated in the future. The potential modifications on the natural milk microbiota composition may have biological implications for infant colonization and metabolism and also, for the development and maturation of the immune and neuroendocrine systems. Conflicting and controversial results have also been obtained when different research groups have compared the effect of the same factor. This can be explained, at least partially, by differences in milk collection and storage procedures, DNA extraction and gene amplification protocols, DNA sequencing methods and bioinformatics analysis, among other factors [3]. International and collaborative research, sharing common protocols from recruitment criteria to bioinformatics, is required in order to enable the comparison of results among research groups and to evaluate the actual composition of the human milk microbiota and microbiome [3,28,29].

In relation to bioinformatics, around the time in which our samples were being collected and analyzed, some articles describing the use of the Illumina application for the bioinformatic analysis of metataxonomic data were published [30,31], and we decided to apply such an approach to our work. The Illumina workflow uses a proprietary algorithm for paired-end reads and has the advantages of being an automated and user-friendly method linked to the highly used Illumina platform, offering a rapid and easy-to-use approach, even for those groups that are not familiarized with bioinformatics, which remains a bottleneck for microbiome studies. However, such application has also relevant limitations, including the impossibility of introducing modifications in the pipeline, the use of a quite limited sequence reference database, and the possibility of including in the analysis a relatively high percentage of interference sequences in low biomass samples, such as those analyzed in our work (milk samples from healthy women). In order to minimize such a possibility, genomic DNA from the PhiX phage was added to the samples in order to assess the correct sequencing; in addition, the R library “decontam” was used to find contaminant sequences and the analyses were focused on the majority of phyla and genera. This approach allowed the obtaining of valuable data and, in fact, most of the most frequent and abundant sequences belonged to bacterial genera that can be isolated from human milk while most sequences related to bacteria typically contaminating molecular biology reagents and kits could be avoided [32]. However, after assessing the pros and cons of the Illumina application, other approaches, such as studying amplicon sequence variants (ASVs) using the Quantitative Insights into Microbial Ecology (Qiime2) and Divisive Amplicon Denoising Algorithm 2 (DADA2) analysis pipelines with taxonomic comparisons to the SILVA 138 database, are recommended for future studies focused on any kind of biological samples and, particularly, on those (including human milk) where a low biomass is expected.

## 5. Conclusions

In conclusion, the data presented in this study indicate that differences in the microbial communities of the human milk were mostly related to the individual woman rather than to the sample collection method, i.e., manual or pumping using single-use sterile collection containers. In addition, no evidence was found that the skimming procedure might introduce a bias into the milk microbial profile. Further studies are needed to confirm the differences found when the sample collection was performed using women’s own containers and pumps or single-use sterile container and an electrical breast pump.

## Figures and Tables

**Table 1:** Bacterial counts (log_10_ cfu/mL) in the milk samples obtained either by manual expression or pumping in this study and identification of the diverse types of colonies observed in the different agar media where growth was observed.

Woman	Sample Collection ^1^	Agar Media ^2^	Bacterial Counts ^3^	Bacterial Species ^4^
W_1	Manual	CNA	3.72 ± 0.13	*S. epidermidis; Corynebacterium* spp.
		MRS-Cys	3.38 ± 0.06	*S. epidermidis; Lc. lactis*
		MCK	3.30 ± 0.13	*A. ursingii*
	Pump	CNA	3.51 ± 0.04	*S. epidermidis; Corynebacterium* spp.
	(SUD)	MRS-Cys	3.34 ± 0.16	*S. epidermidis; Lc. lactis*
		MCK	3.86 ± 0.08	*A. johnsonii*
W_2	Manual	CNA	3.00 ± 0.04	*S. epidermidis*
		MRS-Cys	3.47 ± 0.14	*S. epidermidis*
		MCK	n.d. ^5^	-
	Pump	CNA	3.08 ± 0.11	*S. epidermidis*
	(SUD)	MRS-Cys	2.98 ± 0.11	*S. epidermidis*
		MCK	n.d.	-
W_3	Manual	CNA	n.d.	-
		MRS-Cys	n.d.	-
		MCK	n.d.	-
	Pump	CNA	n.d.	-
	(SUD)	MRS-Cys	n.d.	-
		MCK	n.d.	-
W_4	Manual	CNA	n.d.	-
		MRS-Cys	n.d.	-
		MCK	n.d.	-
	Pump	CNA	2.34 ± 0.10	*S. epidermidis*
	(SUD)	MRS-Cys	2.30 ± 0.11	*S. epidermidis*
		MCK	n.d.	-
W_5	Manual	CNA	2.60 ± 0.16	*S. epidermidis*
		MRS-Cys	3.00 ± 0.16	*S. epidermidis*
		MCK	n.d.	-
	Pump	CNA	2.78 ± 0.11	*S. epidermidis*
	(SUD)	MRS-Cys	2.60 ± 0.10	*S. epidermidis*
		MCK	n.d.	-
W_6	Manual	CNA	n.d.	-
		MRS-Cys	2.64 ± 0.20	*S. epidermidis; Str. parasanguinis/lactarius; L. salivarius*
		MCK	n.d.	*-*
	Pump	CNA	2.86 ± 0.07	*S. epidermidis; Str. parasanguinis/lactarius*
	(SUD)	MRS-Cys	2.58 ± 0.08	*S. epidermidis; L. salivarius*
		MCK	n.d.	-
W_7	Manual	CNA	3.48 ± 0.16	*S. epidermidis; Ko. kristinae*
		MRS-Cys	3.31 ± 0.13	*S. epidermidis; Li. reuteri*
		MCK	n.d.	-
	Pump	CNA	3.33 ± 0.17	*S. epidermidis; Ko. kristinae*
	(SUD)	MRS-Cys	3.16 ± 0.21	*S. epidermidis; Li. reuteri*
		MCK	n.d.	-
W_8	Manual	CNA	3.12 ± 0.21	*S. epidermidis; Str. salivarius*
		MRS-Cys	2.30 ± 0.13	*S. epidermidis*
		MCK	2.79 ± 0.05	*A. ursingii*
	Pump	CNA	4.03 ± 0.11	*S. epidermidis; Str. salivarius*
	(OMP)	MRS-Cys	3.88 ± 0.12	*S. epidermidis*
		MCK	2.26 ± 0.07	*A. ursingii*
W_9	Manual	CNA	2.76 ± 0.07	*S. epidermidis; Str. parasanguinis/lactarius; Ko. rhizophila*
		MRS-Cys	2.60 ± 0.16	*S. epidermidis*
		MCK	n.d.	-
	Pump	CNA	3.26 ± 0.14	*S. epidermidis; Str. parasanguinis/lactarius; Ko. rhizophila*
	(SUD)	MRS-Cys	2.30 ± 0.08	*S. epidermidis*
		MCK	n.d.	-
W_10	Manual	CNA	n.d.	-
		MRS-Cys	n.d.	-
		MCK	n.d.	-
	Pump	CNA	4.38 ± 0.18	*Stn. maltophilia*
	(OMP)	MRS-Cys	n.d.	-
		MCK	4.87 ± 0.09	*K. oxytoca; Stn. maltophilia*

^1^ SUD, Single-use device; OMP, own mother’s pump. ^2^ CNA, Columbia Nadilixic Acid Agar; MRS-Cys, De Man, Rogosa and Sharpe with L-Cysteine agar; MCK, MacConkey agar. ^3^ Data are means of three independent analysis ± standard deviations. ^4^ *A*., *Acinetobacter*; *K*., *Klebsiella*; *Ko*., *Kocuria*; *Lc*., *Lactococcus*; *L*., *Ligilactobacillus* (previously known as *Lactobacillus)*; *Li*., *Limosilactobacillus* (previously known as *Lactobacillus*) *S*., *Staphylococcus*; *Stn*., *Stenotrophomonas*; *Str*., *Streptococcus*. ^5^ n.d.—not detected.

**Table 2:** Bacterial counts (log_10_ cfu/mL) in the aliquots of whole milk and skimmed milk from the samples obtained from women 11 to 20 in this study and identification of the diverse types of colonies observed in the different agar media where growth was observed.

Woman	Agar Media ^1^	Fraction	Bacterial Counts ^2^	Bacterial Species ^3^
W_11	CNA	Whole milk	2.48 ± 0.16	*S. epidermidis*
		Skimmed milk	2.46 ± 0.11	*S. epidermidis*
	MRS-Cys	Whole milk	2.30 ± 0.18	*S. epidermidis*
		Skimmed milk	2.34 ± 0.16	*S. epidermidis*
W_12	CNA	Whole milk	2.57 ± 0.17	*S. epidermidis; Str. salivarius*
		Skimmed milk	2.66 ± 0.10	*S. epidermidis; Str. salivarius*
W_12	MRS-Cys	Whole milk	2.60 ± 0.08	*S. epidermidis*
		Skimmed milk	2.70 ± 0.11	*S. epidermidis*
W_13	CNA	Whole milk	n.d. ^4^	-
		Skimmed milk	n.d.	-
	MRS-Cys	Whole milk	n.d.	-
		Skimmed milk	n.d.	-
W_14	CNA	Whole milk	2.58 ± 0.13	*S. epidermidis*
		Skimmed milk	2.54 ± 0.17	*S. epidermidis*
	MRS-Cys	Whole milk	2.42 ± 0.14	*S. epidermidis*
		Skimmed milk	2.38 ± 0.16	*S. epidermidis*
W_15	CNA	Whole milk	2.38 ± 0.13	*Str. mitis; K. kristinae*
		Skimmed milk	2.67 ± 0.14	*Str. mitis; K. kristinae*
	MRS-Cys	Whole milk	2.56 ± 0.11	*S. epidermidis; L. salivarius*
		Skimmed milk	2.69 ± 0.13	*S. epidermidis; L. salivarius*
W_16	CNA	Whole milk	2.70 ± 0.14	*S. epidermidis; Corynebacterium* spp.
		Skimmed milk	2.73 ± 0.18	*S. epidermidis; Corynebacterium* spp.
	MRS-Cys	Whole milk	2.51 ± 0.16	*S. epidermidis*
		Skimmed milk	2.40 ± 0.21	*S. epidermidis*
W_17	CNA	Whole milk	2.15 ± 0.11	*S. epidermidis; E. faecium*
		Skimmed milk	2.04 ± 0.06	*S. epidermidis; E. faecium*
	MRS-Cys	Whole milk	n.d.	*-*
		Skimmed milk	n.d.	*-*
W_18	CNA	Whole milk	2.72 ± 0.06	*S. epidermidis; R. mucilaginosa*
		Skimmed milk	2.68 ± 0.14	*S. epidermidis; R. mucilaginosa*
	MRS-Cys	Whole milk	2.48 ± 0.11	*S. epidermidis*
		Skimmed milk	2.44 ± 0.13	*S. epidermidis*
W_19	CNA	Whole milk	2.40 ± 0.17	*S. epidermidis*
		Skimmed milk	2.36 ± 0.10	*S. epidermidis*
	MRS-Cys	Whole milk	2.34 ± 0.16	*S. epidermidis; Lc. lactis*
		Skimmed milk	2.41 ± 0.17	*S. epidermidis; Lc. lactis*
W_20	CNA	Whole milk	n.d.	*-*
		Skimmed milk	n.d.	*-*
	MRS-Cys	Whole milk	n.d.	*-*
		Skimmed milk	n.d.	*-*

^1^ CNA: Columbia Nadilixic Acid Agar; MRS-Cys: De Man, Rogosa and Sharpe with l-Cysteine agar. ^2^ Data are means of three independent analysis ± standard deviations. ^3^ *E*., *Enterococcus*; *K*., *Kocuria*; *Lc*., *Lactococcus*; *L*., *Ligilactobacillus* (previously known as *Lactobacillus)*; *R*., *Rothia*; *S*., *Staphylococcus*; *Str*., *Streptococcus.* ^4^ n.d., not detected.

**Table 3:** Relative abundance (%), expressed as the median and the interquartile range, of the 20 most abundant genera depending on the milk extraction method (manual expression or pumping).

Phylum/Genus	Manual Extraction		Pumping		
	*n* (%)	Relative Abundance	*n* (%)	Relative Abundance	*p*-Value ^1^
**Firmicutes**					
*Streptococcus*	8 (100%)	23.94 (14.22–42.83)	8 (100%)	37.49 (10.13–55.44)	0.88
*Staphylococcus*	8 (100%)	20.36 (2.74–22.82)	8 (100%)	8.26 (2.37–14.22)	0.16
*Veillonella*	5 (63%)	0.21 (0.00–1.44)	7 (88%)	0.86 (0.25–2.51)	0.40
*Gemella*	4 (50%)	0.10 (0.00–0.40)	6 (75%)	0.48 (0.20–0.68)	0.36
*Paenibacillus*	8 (100%)	0.19 (0.17–0.20)	8 (100%)	0.14 (0.11–0.16)	0.17
*Clostridium*	8 (100%)	0.13 (0.09–0.20)	6 (75%)	0.17 (0.11–0.19)	1.00
*Bacillus*	7 (88%)	0.18 (0.05–0.39)	7 (88%)	0.07 (0.06–0.14)	0.56
*Granulicatella*	1 (13%)	<0.00	4 (50%)	0.03 (0.00–0.27)	0.10
*Leuconostoc*	1 (13%)	<0.00	4 (50%)	0.03 (0.00–0.17)	0.11
**Actinobacteria**					
*Rothia*	8 (100%)	3.27 (0.72–6.51)	8 (100%)	1.60 (1.26-4.30)	0.75
*Corynebacterium*	8 (100%)	0.39 (0.28-–0.73)	7 (88%)	0.16 (0.08-1.04)	0.44
*Cellulomonas*	4 (50%)	0.13 (0.00–0.37)	5 (63%)	0.09 (0.00-0.22)	0.87
*Streptomyces*	4 (50%)	0.09 (0.00–0.26)	5 (63%)	0.13 (0.00-0.17)	0.87
*Actinomyces*	5 (63%)	0.06 (0.00–0.20)	4 (50%)	0.06 (0.00-0.54)	0.87
**Proteobacteria**					
*Acinetobacter*	5 (63%)	0.34 (0.00–2.07)	6 (75%)	0.28 (0.05–1.00)	0.87
*Sphingomonas*	6 (75%)	0.34 (0.05–0.86)	4 (50%)	0.02 (0.00–0.12)	0.21
*Pseudomonas*	6 (75%)	0.09 (0.03–0.42)	7 (88%)	0.58 (0.15–1.18)	0.23
*Erwinia*	2 (25%)	0.00 (0.00–0.05)	3 (38%)	0.00 (0.00–0.13)	0.65
**Unclassified genera**	8 (100%)	19.49 (14.23–27.96)	8 (100%)	15.82 (14.18–28.15)	0.80

^1^ Kruskal–Wallis rank tests.

**Table 4:** Relative abundance of the most abundant genera in the different milk fractions (whole milk, fat layer and skimmed milk) from milk samples (*n* = 7).

Phylum/Genus	Whole Milk		Fat Layer		Skimmed Milk		
	*n* (%)	Relative Abundance ^1^	*n* (%)	Relative Abundance	*n* (%)	Relative Abundance	*p*-Value ^2^
**Firmicutes**							
*Staphylococcus*	7 (100%)	12.10 (7.95–25.17)	7 (100%)	3.43 (1.23–16.61)	7 (100%)	9.61 (0.94–21.08)	0.53
*Streptococcus*	7 (100%)	2.31 (1.29–4.57)	7 (100%)	4.03 (2.54–18.01)	7 (100%)	2.66 (2.33–4.95)	0.59
*Lactobacillus*	7 (100%)	1.43 (0.78–2.14)	7 (100%)	1.87 (0.86–3.78)	7 (100%)	2.20 (0.46–3.55)	0.95
*Pediococcus*	7 (100%)	1.31 (0.87–1.84)	6 (86%)	1.38 (0.78–1.49)	6 (86%)	1.39 (0.66–1.85)	0.82
*Bacillus*	6 (86%)	0.34 (0.24–0.45)	4 (57%)	0.17 (0.00–0.29)	5 (71%)	0.33 (0.16–0.44)	0.39
*Peptoniphilus*	5 (71%)	0.35 (0.11–1.44)	5 (71%)	0.81 (0.40–1.27)	3 (43%)	0.00 (0.00–0.84)	0.56
*Lactococcus*	3 (43%)	0.00 (0.00–2.91)	4 (57%)	0.22 (0.00–6.98)	6 (86%)	3.57 (1.76–7.09)	0.33
**Actinobacteria**							
*Propionibacterium*	5 (71%)	1.11 (0.12–4.98)	6 (86%)	6.52 (0.60–7.08)	6 (86%)	1.37 (1.14–2.35)	0.84
*Corynebacterium*	5 (71%)	1.11 (0.23–2.52)	3 (43%)	0.00 (0.00–0.84)	4 (57%)	0.48 (0.00–3.13)	0.57
*Bifidobacterium*	2 (29%)	0.00 (0.00–0.66)	3 (43%)	0.00 (0.00–0.33)	2 (29%)	0.00 (0.00–0.14)	0.84
**Proteobacteria**							
*Chondromyces*	5 (71%)	2.51 (0.99–6.15)	5 (71%)	3.43 (1.23–3.80)	4 (57%)	1.31 (0.00–4.18)	0.79
*Burkholderia*	7 (100%)	0.77 (0.64–0.86)	7 (100%)	1.40 (1.06–1.60)	6 (86%)	1.03 (0.62–1.12)	0.04
*Sphingomonas*	5 (71%)	0.60 (0.12–3.32)	6 (86%)	0.94 (0.41–1.46)	4 (57%)	0.38 (0.00–0.75)	0.38
*Hyphomicrobium*	5 (71%)	0.30 (0.11–0.68)	6 (86%)	0.43 (0.30–0.62)	3 (43%)	0.00 (0.00–0.37)	0.28
*Novosphingobium*	2 (29%)	0.00 (0.00–0.21)	3 (43%)	0.00 (0.00–0.37)	4 (57%)	0.13 (0.00–0.52)	0.69
*Azospirillum*	2 (29%)	0.00 (0.00–0.18)	4 (57%)	0.38 (0.00–0.68)	4 (57%)	0.43 (0.00–1.47)	0.25
*Chondromyces*	5 (71%)	2.51 (0.99–6.15)	5 (71%)	3.43 (1.23–3.80)	4 (57%)	1.31 (0.00–4.18)	0.79
**Bacteroidetes**							
*Pedobacter*	7 (100%)	0.61 (0.38–0.88)	7 (100%)	0.56 (0.42–0.83)	6 (86%)	0.72 (0.44–0.89)	0.96
**Unclassified genera**	7 (100%)	27.7 (23.86–31.55)	7 (100%)	31.81 (28.09–34.53)	7 (100%)	30.82 (26.71–35.82)	0.66

^1^ The relative abundance (%) of each bacterial genus is expressed as the median and the interquartile range. ^2^ Kruskal–Wallis rank tests.

## Supplementary Materials

The following are available online at https://www.mdpi.com/2076-2607/8/9/1278/s1, Figure S1: Alpha diversity in human milk samples obtained with different collection methods; Figure S2: Alpha diversity in human milk samples according to their processing.
