# Supplementary material for: Effect of Sample Collection (Manual Expression vs. Pumping) and Skimming on the Microbial Profile of Human Milk Using Culture Techniques and Metataxonomic Analysis
Source: Microorganisms. 2020 Aug 21;8(9):1278. doi: 10.3390/microorganisms8091278 (PMC7564974; doi:10.3390/microorganisms8091278)
Supplement: Supplementary file 1 [file microorganisms-08-01278-s001.pdf]

microorganisms

Article

Effect of Sample Collection (Manual Expression vs. Pumping) and Skimming on the Microbial Profile of Human Milk Using Culture Techniques and Metataxonomic Analysis

Maricela Rodríguez-Cruz [1,2,*], Claudio Alba [3], Marina Aparicio [2], María Ángeles Checa [4], Leonides Fernández [3] and Juan Miguel Rodríguez [2,*]

[1] Laboratorio de Nutrición Molecular, Unidad de Investigación Médica en Nutrición, Hospital de Pediatría, Centro Médico Nacional Siglo XXI. Instituto Mexicano del Seguro Social. Av. Cuauhtémoc No. 330, Col Doctores, Delegación Cuauhtémoc, 06725 Mexico City, Mexico

[2] Department of Nutrition and Food Science, Faculty of Veterinary Sciences,Complutense University of Madrid, 28040 Madrid, Spain; marinaap@ucm.es

[3] Department of Galenic Pharmacy and Food Technology, Faculty of Veterinary Sciences, Complutense University of Madrid, 28040 Madrid, Spain; claudio.alba@ucm.es (C.A.), leonides@ucm.es (L.F.)

[4] Centro de Salud Arrabal, 50015 Zaragoza, Spain; manchecadiez@gmail.com

* Correspondence: maricela.rodriguez.cruz@gmail.com (M.R.-C.); jmrodrig@ucm.es (J.M.R.); Tel.: +34-91394-3837 (J.M.R.)

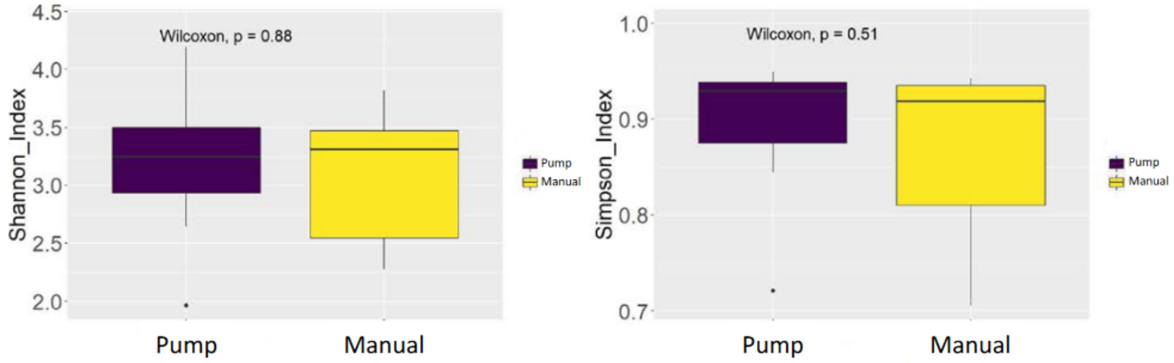

Supplementary Figure A1. Alpha diversity in human milk samples obtained with different collection methods. The box-plots illustrate the alpha diversity indices (Shannon diversity and Simpson index) in human milk samples collected from women 1 to 8 by pumping with single-use sterile devices or by manual expression. Median (horizontal line in the box) and interquartile ranges (vertical lines or whiskers extending from the top and bottom of the box) are indicated in the plots. No significant differences between samples according to the collection method ($p > 0.05$; Wilcoxon rank sum tests).

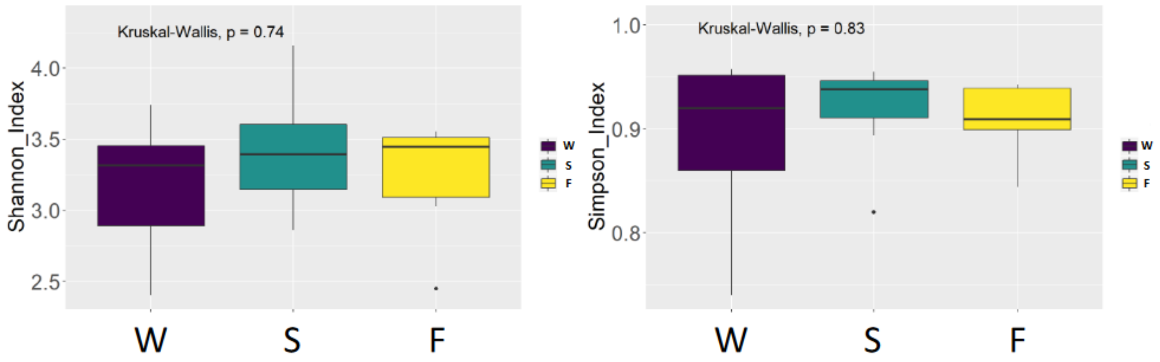

Supplementary Figure A2. Alpha diversity in human milk samples according to their processing. The box-plots illustrate the alpha diversity índices (Shannon diversity and Simpson index) in whole human milk samples (W) collected from women 21 to 27 and their fractions: the skimmed milk (S) and the fat layer (F). Median (horizontal line in the box) and interquartile ranges (vertical lines or whiskers extending from the top and bottom of the box) are indicated in the plots. No significant differences between samples according to the collection method (p > 0.05; Kruskal-Wallis tests)
